# Detecting Awareness in the Vegetative State: Electroencephalographic Evidence for Attempted Movements to Command

**DOI:** 10.1371/journal.pone.0049933

**Published:** 2012-11-21

**Authors:** Damian Cruse, Srivas Chennu, Davinia Fernández-Espejo, William L. Payne, G. Bryan Young, Adrian M. Owen

**Affiliations:** 1 Brain and Mind Institute, University of Western Ontario, London, Ontario, Canada; 2 Department of Clinical Neurosciences, University of Cambridge, Cambridge, United Kingdom; 3 Parkwood Hospital, London Health Sciences Centre, London, Ontario, Canada; 4 Department of Clinical Neurological Sciences, University of Western Ontario, London, Ontario, Canada; University College London, United Kingdom

## Abstract

Patients in the Vegetative State (VS) do not produce overt motor behavior to command and are therefore considered to be unaware of themselves and of their environments. However, we recently showed that high-density electroencephalography (EEG) can be used to detect covert command-following in some VS patients. Due to its portability and inexpensiveness, EEG assessments of awareness have the potential to contribute to a standard clinical protocol, thus improving diagnostic accuracy. However, this technique requires refinement and optimization if it is to be used widely as a clinical tool. We asked a patient who had been repeatedly diagnosed as VS for 12-years to try to move his left and right hands, between periods of rest, while EEG was recorded from four scalp electrodes. We identified appropriate and statistically reliable modulations of sensorimotor beta rhythms following commands to try to move, which could be significantly classified at a single-trial level. These reliable effects indicate that the patient attempted to follow the commands, and was therefore aware, but was unable to execute an overtly discernable action. The cognitive demands of this novel task are lower than those used previously and, crucially, allow for awareness to be determined on the basis of a 20-minute EEG recording made with only four electrodes. This approach makes EEG assessments of awareness clinically viable, and therefore has potential for inclusion in a standard assessment of awareness in the VS.

## Introduction

Conventional assessments of consciousness following severe brain-injury, such as the Glasgow Coma Scale [1] and the Coma Recovery Scale - Revised (CRS-R [2]), rely on overt motor responses to command in order for a patient to be considered to be aware. Those patients who appear to be awake, but who show no external evidence of awareness on the basis of these behavioral assessments, are considered to be in a Vegetative State (VS [3]–[5] ). However, it is a challenge for clinicians to identify appropriate responses to command as these may be only minimal or inconsistently present. Indeed, this difficulty is thought to be a primary contributing factor to the ∼40% misdiagnosis rate for VS [6]–[8]. In recent years, however, it has become increasingly apparent that the absence of behavioral evidence for command-following is not necessarily indicative of the true absence of awareness, or of the absence of an ability to follow commands under appropriate conditions [9]–[13].

Owen et al. [11] re-characterized the way in which an individual can be said to respond to command, by including the hemodynamic response of the brain, as detected with functional magnetic resonance imaging (fMRI). In that study, a patient who appeared to be in a VS was asked to perform two mental imagery tasks – imagining playing tennis and imagining walking through the rooms of her house – that are associated with the differential activation of a number of distinct brain regions. The resulting patterns of activity were entirely comparable with those observed in healthy, awake participants performing these same imagery tasks to command. These results allowed Owen et al. to conclude that the patient was responding to command and therefore retained a level of awareness that was not apparent from her (lack of) behavior. In spite of these advances, however, fMRI does not provide a viable means of assessing covert awareness on a routine basis. Alongside considerations of cost and scanner availability, the physical stress incurred by patients as they are transferred to a suitable facility is significant.

Electroencephalography (EEG), on the other hand, is considerably less expensive than fMRI and is entirely portable. When an individual imagines or plans a movement of a limb, sensorimotor cortical activity is reflected in the scalp EEG as changes in the power of oscillations in the mu and/or beta frequency bands (∼7–30 Hz) over central electrodes. Typically, these are manifest as reductions in power – or event-related desynchronizations (ERD) – over the scalp contralateral to the limb, and increases in power – or event-related synchronizations (ERS) – over the ipsilateral scalp [14]–[16]. Cruse et al. [9] (see also [10]) exploited these patterns of EEG activity in order to identify statistically reliable covert command-following in 19% of 16 VS patients who were asked to imagine moving their right-hands and their toes. Crucially, this was determined for the first time at the patients’ bedsides. EEG systems are portable, relatively inexpensive, and available in many hospitals. Consequently, EEG-based assessments of awareness may be included in standard clinical assessments with minimal difficulty. However, before this can be widely deployed, the EEG approach to detecting awareness must be optimized for the clinical setting.

In their original study, Cruse et al. [9] employed EEG recordings from up to 257 scalp electrodes. However, a typical clinical setting will not have access to such state-of-the-art equipment, and will have only 20 or fewer channels [17]. Therefore, in order for EEG assessments to become a standard approach, they must return reliable results with only a minimal, clinical EEG setup.

For any assessment to be clinically viable it must also maximize the likelihood of detecting awareness when it is present – i.e. true positives. The successful completion of the motor imagery task employed by Cruse et al. [9] required many high-level cognitive faculties, including the ability to hold the task instructions in mind across a delay of up to 90-seconds. While this requirement allowed Cruse et al. to draw strong conclusions regarding the high level of cognitive functioning retained by those patients who returned positive results, it is also likely to have excluded a proportion of patients who were aware, but lacked sufficient cognitive resources to complete the task, hence resulting in false negatives. Indeed, during conventional behavioral assessments of awareness, a considerably less cognitively demanding, though entirely motoric, output is required. Under the guidelines for the CRS-R, the international standard for the diagnosis of VS, a patient is given a simple auditory instruction to try to move their hand and provided with a brief period of time in which to perform this action. When a successful movement to command occurs on 3 out of 4 trials, the patient is diagnosed as ‘aware’, and therefore not VS. For the same conclusion to be drawn based on EEG-detected covert command-following, therefore, a similarly simple task may be sufficient. Accordingly, Bekinschtein et al. [18] instructed VS patients to try to move their hands in the fMRI scanner, and observed appropriate premotor activity in two patients, despite the absence of an overtly discernable motor output. The success of this approach therefore indicates that functional neuroimaging can be used to detect *attempts* to follow command, as indexed by the appropriate premotor activity, even when these attempts are not executed in a behaviorally discernable way.

Clinical assessments must also minimize the likelihood of false positives – i.e. the chances of apparently detecting command-following when none has occurred. In the absence of an accurate estimate of false negative rates for motor imagery in the patient population, the absence of a positive result is not automatically a ‘negative’ finding, but a ‘null’ result. Since these can occur even in healthy aware individuals due to the less than perfect sensitivity of functional neuroimaging methods, null results are entirely inconclusive with regards to the presence of awareness in the VS. Since an inconclusive result is less likely to lead to changes in the care of a patient, relative to a statistically verifiable positive result, it is typical to place an emphasis on minimizing false positives over null results. In order to estimate the likelihood of false positives, previous studies have contrasted the results of healthy individuals who listen to the task instructions and are asked to follow the commands, with those same individuals who listen to the instructions and are asked to *not* follow the commands [9], [19]. When no positives are returned in the latter case, the test can be considered to minimize the likelihood of false positives.

Here we describe a clinically viable approach to detecting awareness in the VS. While EEG was recorded from a minimal set of electrodes, we instructed a patient who had been diagnosed as VS for more than 12-years to try to move his left and right hands. We analyzed the data both at a traditional trial-average level, and at a single trial level (as [9], [10]). The average-level analyses identify significant clusters of time-varying ERDs and ERSs, while the single-trial analyses provide a measure of the consistency of these responses following each individual instruction. A group of 6 healthy control participants also completed the task in the way described above in order to estimate the likelihood of null results and false positives.

## Methods

### Ethics Statement

The patient’s surrogate decision makers gave informed written consent for his participation. The Health Sciences Research Ethics Board of the University of Western Ontario provided ethical approval for the patient study. Healthy participants gave informed written consent. The Psychology Research Ethics Board of the University of Western Ontario provided ethical approval for the healthy study.

### Patient

The patient was a 38-year old male who had been repeatedly diagnosed as VS for 12-years following a traumatic brain injury sustained in a road-traffic incident. On the day of his EEG assessment, his CRS-R diagnosis was VS with a score of 6: (auditory function: startle; visual function: none; motor function: flexion withdrawal; oromotor/verbal function: oral reflexive movement; communication: none; arousal: eye opening without stimulation). The research team had behaviorally assessed the patient with the CRS-R eight times across the three months prior to his EEG assessment, and had always returned a diagnosis of VS (CRS-R score range 4–7).

### Healthy Controls

Six healthy participants (all male, median age 29, range 22–32) gave informed consent and served as the control group.

### Procedure

Each trial began with one of three instructions: ‘Try to move your right-hand’, ‘Try to move your left-hand’, and ‘And now, relax’. All instructions were 2-seconds in length and were followed by between 4- and 7-seconds of silence (selected randomly from a uniform distribution on each trial) before the onset of the next instruction. The instructions were presented by earphone. The task was completed in blocks of 36 trials (12 × each instruction) presented in a pseudorandom order so that no more than three instructions of the same type were presented consecutively. The patient completed five blocks during the assessment, for a total of 180 trials, with short breaks between each in order to reduce fatigue.

All healthy controls completed the same task as the patient in which they rapidly squeezed their hands into a fist four times following the instructions to move. In a separate run, control participants also listened to the same task but were instructed simply to mind-wander rather than to follow the commands. Order of task completion was counter-balanced across healthy participants.

### EEG Recording and Pre-Processing

EEG was recorded using the g.Gamma active electrode system (g.tec Medical Engineering, Austria) from 9-electrodes at FC3, FCz, FC4, C3, Cz, C4, CP3, CPz, and CP4, housed in an electrode cap. Due to the expected laterality of EEG responses, and the success of previous studies [20], only data from the 4 non-midline electrodes were used for subsequent analysis (FC3, FC4, CP3, CP4). Data were sampled at 256 Hz and referenced to the right earlobe, with impedances kept below 5kohms. EEG data were subsequently down-sampled to 100 Hz and filtered offline between 1 and 40 Hz. Specifically, the EEGLAB function ‘pop_eegfilt’ was used to perform two-way least-squares finite impulse response filtering, first with a high-pass cutoff of 1 Hz, and then with a low-pass cutoff of 40 Hz. The data were subsequently segmented into 6-second epochs time-locked to the onset of each instruction (or equivalently, up to 4 seconds after the offset of the instruction). Trials containing muscular artifacts were identified by visual inspection and removed. For the patient, after artifact-rejection, 36 left-hand trials, 33 right-hand trials, and 34 rest trials contributed to the analyses (for a total of 103 trials, or 57% of trials). In order to make the healthy data as comparable as possible, the same numbers of clean trials were included in all healthy analyses as those that contributed to the patient analyses. Data were re-referenced offline to form two bipolar channels (FC3-CP3, FC4-CP4) that shall be referred to as C3’ and C4’, respectively. As compared to mastoid referencing, this bipolar approach is known to detect sensorimotor mu and beta modulations with a high level of accuracy across a large proportion of healthy individuals, due to the smaller contribution of distal EEG sources to the bipolar signal [20].

### EEG Average Spectral Analysis

Spectral power estimates were calculated for each time-point at C3’ and C4’ separately using a fixed-window (1-second, Hanning window) time-frequency transformation (FieldTrip [21] ‘ft_freqstatistics’ function). The time-frequency data at each electrode was subsequently compared for left-hand vs. rest and right-hand vs. rest. The statistical significances of these differences were computed by means of cluster-based permutation testing which inherently controls for multiple comparisons. Briefly, the statistical procedure is as follows. For every time-frequency sample the experimental conditions are compared by a t-test. All samples for which the t-test value is significant at p<.05 (two-tailed) are selected and clustered into groups based on their temporal and spectral adjacency, and the sum of t-test values for each cluster is calculated (with no lower or upper limits on cluster size). The statistical significance of each cluster is then determined by means of a Monte Carlo randomization test in which the original trials are randomly partitioned (removing task-related differences) and the procedure above is repeated 1000 times. The maximum cluster sums from each repetition form a surrogate distribution to test the null hypothesis that each cluster comes about by chance. The significance threshold for the cluster statistics was set to.05/4 (.0125, two-tailed) to control for the use of four separate cluster analyses (2 comparisons × 2 EEG channels). The full statistical procedure is described in detail by Maris & Oostenveld [22] and was implemented with the open-source MATLAB toolbox FieldTrip [21].

Alongside this analysis, in order to fully visualize the patient’s EEG in a way similar to that of a clinical EEG recording, average power spectra – i.e. non-time-varying spectral power – were also calculated separately for each bipolar channel for each condition. Specifically, the MATLAB function ‘pwelch’ was used to calculate the average power (in dB) across the entire 6-second epoch. This function divides each segment into eight sections with 50% overlap, computes eight periodograms with a Hamming window, and returns the average spectral density across the eight sections.

### EEG Single-Trial Classification Procedure

The spectral analysis above was complemented by single-trial classification of the data. The features employed for classification were log bandpower values in four frequency bands: 7–13 Hz (mu), 13–19 Hz (low-beta), 19–25 Hz (mid-beta), and 25–30 Hz (high-beta), as employed by Cruse et al. [9], [10]. Spectral power in each band was estimated using a short-time Fourier transform (MATLAB function ‘spectrogram’) with a sliding window of 1-second (as recommended by [23]) moving in 50 ms steps. This resulted in four bandpower values at each of 100 time-points per trial per channel (i.e. four frequency bands in 50 ms increments from 1000 ms before the offset of the instruction to 3950 ms after the offset of the instruction).

Classification was performed between left-hand and rest, and right-hand and rest, at each time-point separately using the 8 log bandpower features (2 channels × 4 frequency bands). In order to estimate the classifier’s generalizability, 10-fold cross-validation was performed. Specifically, the trials were separated into 10 approximately equal-sized groups. Due to its low computational demands, a naïve Bayes classifier was trained on the features from 9 of these groups – the training set (using MATLAB’s ‘naivebayes’ object). The classes of each of the trials in the remaining group – the test set – were then predicted using this model in order to calculate the classifier’s accuracy. Briefly, a naïve Bayes classifier estimates the parameters of a probability distribution (in this case, the mean and standard deviation of a normal distribution) per training feature per class. By applying Bayes’ Theorem, the test sample is used to calculate posterior probabilities for each class and is subsequently classified into the class with the highest posterior probability (for further details on naïve Bayes classification, see [24]). This was repeated 10-times with a different test set each time, so that each trial was tested exactly once. The average classification accuracy across the 10 cross-validation folds was then calculated at each time-point. This resulted in an accuracy time-course that mirrored the dynamics of the spectral ERD/ERS. In order to control for outliers, and to return a statistically robust estimate of classification accuracy, this time-course was then smoothed with a sliding-window of 500 ms.

The classification accuracies were evaluated to be statistically significant by means of a familywise randomization test with 1000 repetitions [25]. Specifically, at each permutation the class labels of the trials were randomly shuffled in order to remove any systematic differences, and the above cross-validated classification procedure was repeated. At the end of each repetition, the best smoothed accuracy across all time-points was recorded in order to jointly control the familywise error rate over time-points. The maximum accuracies calculated over the repetitions formed a surrogate distribution representing the null hypothesis that the classifier was operating at chance. The participant’s original accuracy was then evaluated against this distribution to calculate a p-value at each time-point separately. A significance threshold of.05/2 (.025, one-tailed) was employed to control for the two independent comparisons (left-hand vs. rest, and right-hand vs. rest).

Therefore, the average spectral analysis and the single-trial classification procedure make use of the same type of measures derived from the data – i.e. time-frequency estimates. The only difference is that the average spectral analysis employs 1 Hz resolution in order to identify the minutiae of spectral variation, while the single-trial analysis uses a coarser frequency resolution (4 bands from 7–30 Hz), in order to maintain a low number of features for classification, as well as for consistency with previous research [9].

All calculations were performed with MATLAB, using a combination of custom scripts and EEGLAB [26] and Fieldtrip [21] functions.

## Results

### Average Spectral Analyses

**Figure 1:**
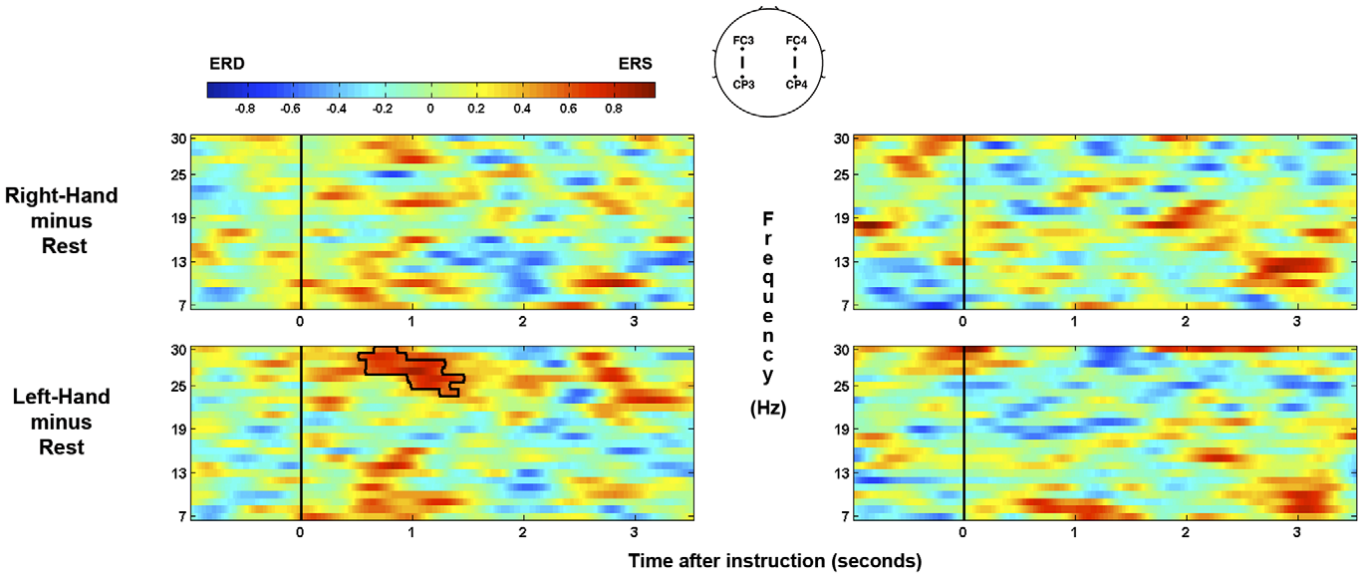
Time-frequency plots for right-hand and left-hand trials for the patient. The significant ipsilateral high-beta ERS cluster is highlighted. Plots on left and right reflect left- and right-hemisphere EEG channels (C3’ and C4’ respectively). Time is measured relative to the offset of the verbal instructions. Color scale denotes the log ratio of power versus rest.

For the patient, significant spectral power differences are shown in Figure 1 for each comparison of conditions at left-hemisphere (FC3-CP3: C3’) and right-hemisphere (FC4-CP4: C4’) separately. As can be seen, when compared with rest, a significant ipsilateral ERS occurred in the high-beta band ∼1-second after the offset of the instruction to move the left-hand. There were no significant clusters in the comparison between right-hand and rest. At no point was any behavioral response observed that would be clinically indicative of awareness.

**Figure 2:**
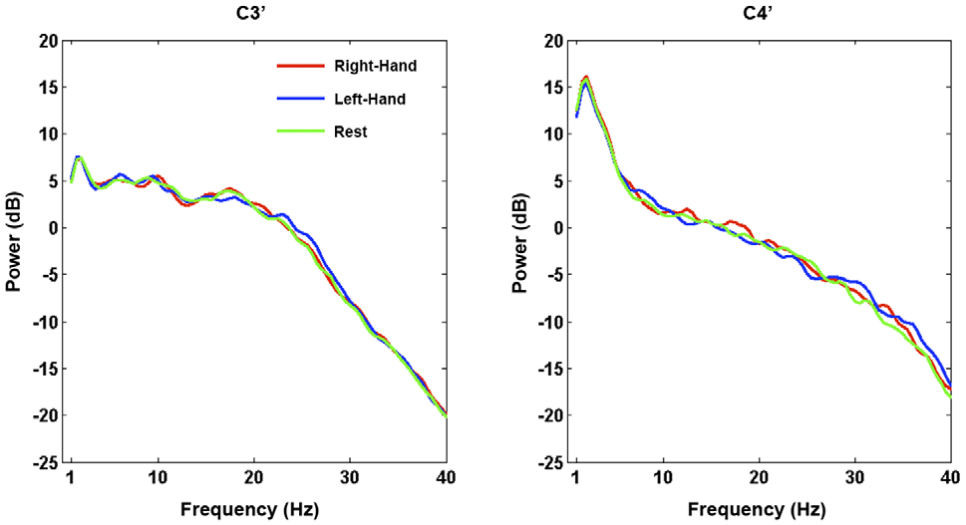
Average spectral power (dB) calculated across all of the patient’s EEG. Data are plotted for each condition at each bipolar channel separately. A right-lateralised delta peak can be seen, with no alpha peak evident.

Figure 2 shows the average power – i.e. non-time-varying – in each condition at each electrode from the patient’s data. As can be seen, his overall EEG contains high power in the delta band over the right-hemisphere, with no alpha peak evident. Importantly, there are no broadband differences between conditions at either electrode that may suggest the presence of artifacts.

**Figure 3:**
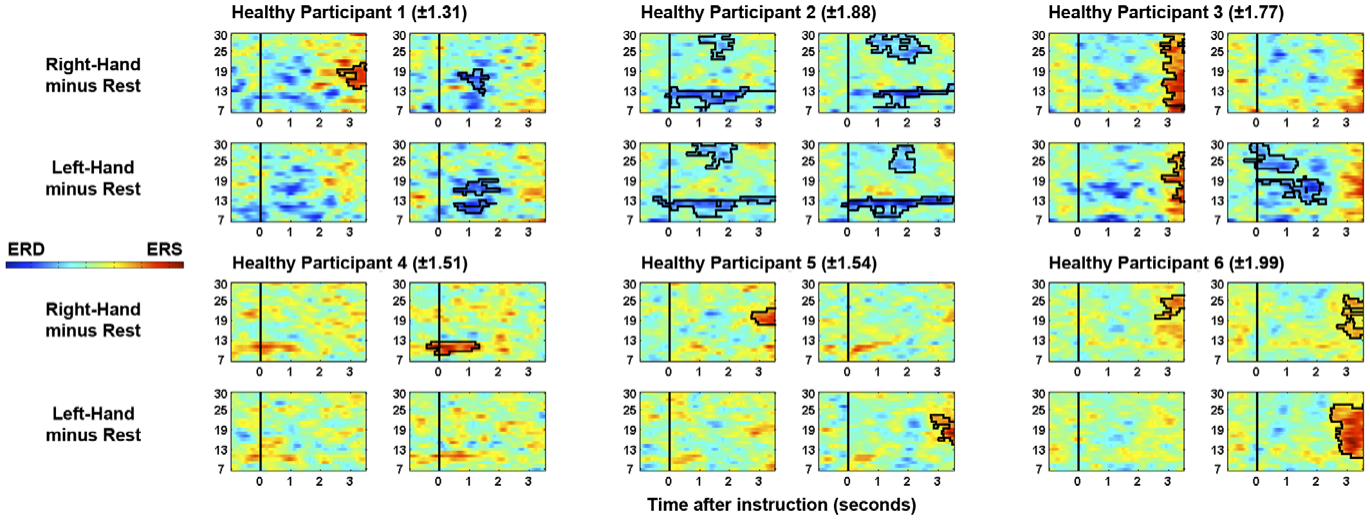
Time-frequency plots for right-hand and left-hand trials for each healthy participant. The range of power values (log ratio versus rest) that are plotted for each healthy control is indicated in parentheses. Significant clusters are highlighted. Plots on left and right for each participant reflect left- and right-hemisphere EEG channels (C3’ and C4’ respectively), as in Figure 1. Time is measured relative to the offset of the verbal instructions. Frequency (Hz) is indicated on the vertical axis.

All healthy participants produced significant post-instruction clusters of ERD and/or ERS in at least one comparison. The specific frequency bands and relative-laterality of ERD/ERS are visualized in Figure 3. As can be seen, individual differences in ERD/ERS are evident across healthy participants, consistent with previous literature (e.g. [15], [27], [28]). Conversely, no significant clusters of ERD or ERS were observed when the same participants were instructed to mind-wander and not to follow the commands.

### Single-trial Analyses

**Figure 4:**
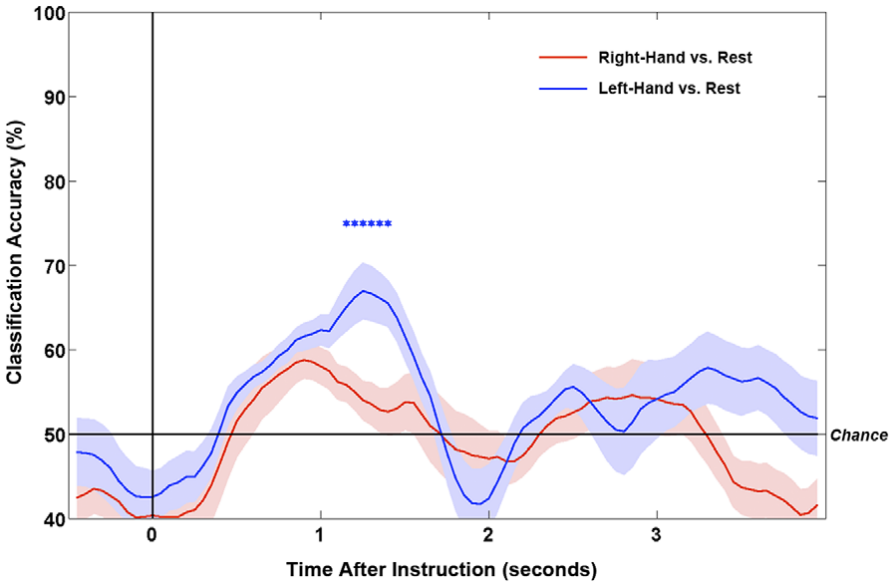
Time-courses of classification accuracies (versus rest) for right-hand and left-hand trials for the patient. Lines show means of the 10-fold smoothed classification accuracies. Shaded areas show ±1 standard errors. Stars denote time-points with significantly above chance classification for left-hand vs. rest (p<.025).

For the patient, the time-courses of smoothed classification accuracies are shown in Figure 4 for the two comparisons. The maximum classification accuracies (smoothed and unsmoothed) and their timings are detailed in Table 1. Only the classification between left-hand and rest was significantly above chance, reaching a smoothed maximum of 67% at 1250 ms after the offset of the instruction.

**Table 1:** Maximum smoothed and unsmoothed classification accuracies for each comparison.

Comparison	Maximum Smoothed Accuracy (Time)	Maximum Unsmoothed Accuracy (Time)
Right-Hand vs. Rest	59% (900 ms)	68% (550 ms)
Left-Hand vs. Rest	67% (1250 ms)*	74% (1150 ms)

Time-point of maximum accuracy, relative to the offset of the instructions, is in parentheses. Statistical significance is denoted with an asterisk (p<.025; significance only calculated on smoothed data).

**Figure 5:**
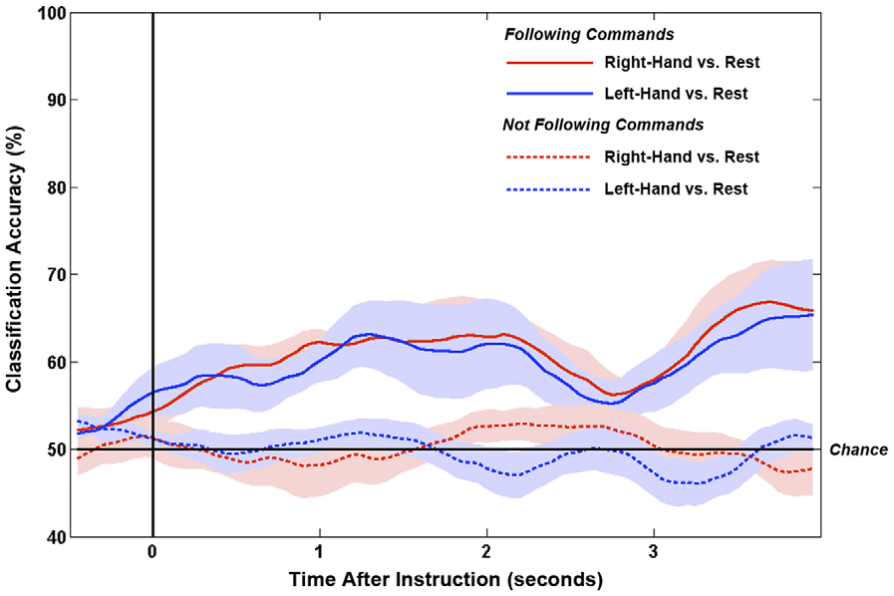
Grand average time-courses of classification accuracies (versus rest) for right-hand and left-hand trials for the healthy control group. Classification time-courses are shown for the trials in which the participants were instructed to follow commands, as well as when instructed to not follow commands. Lines show means of the mean smoothed classification accuracies across participants. Shaded areas show ±1 standard errors.

Five of the six healthy participants returned significantly above chance classification in at least one comparison (median 66.5%, range 60–80%; see Table 2). Conversely, when instructed to mind-wander and not to the follow the commands, no healthy controls returned significant classification in any comparison (median 57%, range 52–63%). The grand average smoothed classification accuracies across healthy controls are shown in Figure 5.

**Table 2:** Maximum smoothed classification accuracies for each comparison for each healthy participant.

	Following Commands		Not Following Commands	
Healthy Participant	Right-Hand vs. Rest	Left-Hand vs. Rest	Right-Hand vs. Rest	Left-Hand vs. Rest
1	68% (1250 ms)*	67% (950 ms)*	54% (200 ms)	57% (200 ms)
2	78% (1350 ms)*	80% (1550 ms)*	63% (650 ms)	62% (950 ms)
3	67% (1300 ms)*	76% (1300 ms)*	58% (1350 ms)	52% (850 ms)
4	66% (400 ms)*	60% (−450 ms)	57% (1750 ms)	55% (3400 ms)
5	62% (0 ms)	61% (0 ms)	54% (700 ms)	54% (550 ms)
6	61% (1400 ms)	66% (2450 ms)*	60% (2400 ms)	60% (2100 ms)

Note how no healthy participant returns significant classification accuracy when not following commands (see Methods). Time-point of maximum accuracy, relative to the offset of the instructions, is in parentheses. Statistical significance is denoted with an asterisk (p<.025).

### Patient Post Hoc Analyses

For the patient, while a statistically significant ERS and classification accuracy were observed following instructions to move his left-hand, no such significant effects were observed for right-hand trials. To investigate this further, we narrowed the frequency band employed in the single-trial classification procedure to only that which produced a significant ERS for left-hand trials (high-beta: 25–30 Hz), and found significantly above chance classifiability for right-hand as well as left-hand trials (versus rest; 65.3% and 66.9% respectively, smoothed).

The above analyses conducted on the bipolar-referenced C3’ and C4’ were also conducted on the mastoid-referenced C3 and C4 and returned similar, though diminished effects, as would be expected (see [20], [29]).

## Discussion

VS patients do not produce any behaviorally discernible evidence that they can follow instructions to move, and are therefore considered to be unaware of themselves and of their environment. However, by recording EEG from only four electrodes while giving the same instruction to move, we have shown that it is possible to detect *attempts* to follow these commands in a VS patient, even if no discernable overt motor output is produced. Crucially this could be detected on a single-trial basis using clinically viable EEG methods at a significantly greater than chance level.

The patient had been diagnosed as VS for 12-years following a traumatic brain injury sustained in a road traffic incident. Indeed, on the day of his EEG assessment, as well as over the three months prior to it, the patient had continually been diagnosed as VS using the international standard assessment tool, the CRS-R. However, from the EEG evidence described here, it is clear that behavioral assessments alone were not sufficient to provide him with an accurate diagnosis.

When the patient was asked to try to move his left-hand during the EEG assessment, a consistent ipsilateral ERS that passed rigorous statistical significance testing was observed in the beta band (relative to when he was asked to rest). The mu and beta rhythms are thought to be idling rhythms of the sensorimotor cortex [30], [31]. When a region of sensorimotor cortex becomes active a reduction in amplitude of these rhythms (an ERD) is observed over the region that is no longer idling, but involved in a motor plan/action. Ipsilateral ERSs during unilateral hand movements/imaginations are considered to reflect the inhibition of the contralateral hand representation [15] and have previously been used to differentiate between motor tasks on a single-trial basis in healthy individuals [20]. Statistically reliable beta ERSs have also been observed in attempted movements of tetraplegic and paraplegic patients [32]. Indeed, tetraplegic patients have been trained to control hand orthoses through the modulation of sensorimotor beta [33], [34]. While the overall EEG of this patient (and indeed VS patients as a whole) contains higher power in low frequencies, such activity is equally distributed across trials of each type (see Figure 2) and therefore not endogenously modulated by the task. Indeed, as with the patient data, two of the six healthy controls also produced significant responses in only beta frequencies (>12 hz; see Figure 3).

The statistically reliable modulations of the patient’s sensorimotor rhythms are therefore consistent with attempts to move. Under this assumption, we could conclude that the patient was attempting to follow the commands. If he were to have successfully followed the commands in a behaviorally identifiable way, he would have been considered to be aware. At the very least, this patient would be diagnosed as in the minimally-conscious state (MCS [2], [4], [35]). The current EEG evidence for attempted movement to command, therefore, should lead us to the same conclusion – that this patient was aware. To further support this, we asked six healthy participants to complete the same task and found significantly reliable patterns of ERD and ERS for all participants, and significantly above chance single-trial classification for five out of six participants (see Results for details). Crucially, when these same participants were asked to listen to the same instructions but not to follow commands, no significant effects were observed in either the average or single-trial analyses for any participant (see Figure 5). These results confirm that simply hearing the task instructions is not sufficient to return a positive EEG result. Rather, an act of (attempted) command-following must occur.

The outcomes of the *post hoc* analyses (see Results) suggest that the patient was also attempting to move his right hand to command – as indicated by classifiable modulations of his high-beta rhythms. However, it is likely that there was too much variability in the modulation of the other frequency bands for the classifier to accurately generalize across trials, or for the average spectral analyses to identify it in the *a priori* analyses. This putative variability may also be the cause of the absence of statistically reliable contralateral ERDs alongside the ipsilateral ERSs. While contralateral mu ERDs are often observed in a range of motor tasks (see [23]), single-subject variability is very high in this regard (e.g. [27], [28], [32], [36]) due to variations in the cognitive strategies employed and neural generators involved ([28], [37]–[39]). Indeed, even within an individual participant, widely varying patterns of motor-related EEG reactivity can be seen across sessions [36]. This variability has led to an inability to detect reliable mu/beta modulations even with machine-learning techniques in some healthy individuals [20]. The absence of a reliable contralateral mu ERD in the patient’s data may therefore reflect differences in the way in which he performed the task, such as the type of movement he attempted to perform [28]. It has also been suggested that the EEG of severely brain-injured patients may react in a different manner to healthy controls during motor tasks due to cortical reorganization that occurred post-injury [40]. Nevertheless, in this regard it is worth noting that two of our six healthy controls also produced significantly classifiable EEG data in only one of the two comparisons. One control only produced a statistically reliable ipsilateral ERS in one comparison, and significant contralateral ERDs were not observed in half of our healthy controls. Figure 3 highlights this well-documented individual variability in the EEG response of the healthy participants, in terms of the significantly-varying frequency band, its time-course, and laterality [15]. The variability in the pattern of responses observed in the patient data is not inconsistent with those of the healthy control group when following the commands. Indeed, the significant responses to command therein manifest despite the potentially large variations in arousal levels documented in VS and MCS patients across both short and long time periods [2], [41]. Nevertheless, the conclusion regarding right-hand movements to command must be made cautiously since the choice of frequency band for that comparison used data from the ‘rest’ condition, which had also been used in the left-hand analyses. As with the guidelines for behavioral assessment, these results therefore highlight the importance of employing both the grand average and single-trial analyses in order to provide patients with every opportunity to demonstrate their covert abilities.

The task employed in the initial proof-of-concept by Cruse et al. [9] required many high level cognitive abilities in order for a positive EEG result to be returned, including the ability to hold task instructions in memory for ∼90-seconds. The current task, however, did not make such cognitive demands on the patient since the movement attempt was to be executed immediately after each instruction. The inclusion of a ‘rest’ condition in the current task also reduced the cognitive load compared with Cruse et al. [9] since this condition did not require an action, but rather the lack of one, which could be compared with the active conditions. While this less demanding task may not provide us with the same insights about the upper extent of cognitive ability retained by this patient, it is however capable of identifying a level of awareness that is not evident from external behavior, and may therefore increase the number of patients who are able to detectably follow command. Nevertheless, the current task does require a number of cognitive faculties, including language comprehension and task switching. In order to reduce the possibility of false-negatives due to these paradigmatic demands, therefore, this EEG approach should ideally be a part of a battery of assessments that probe the range of cognitive abilities extant in a particular patient (e.g. [42], [43]).

The assessment of awareness described here made use of ∼20-minutes of EEG data recorded from only four electrodes. The majority of hospitals worldwide are equipped with the basic EEG hardware that could perform the same recording. Such basic requirements argue strongly for the incorporation of EEG assessments of awareness into the standard clinical assessment following brain injury. Indeed, a resting-state EEG recording is currently performed as part of the standard clinical course following brain injury. The EEG assessment of awareness described here could therefore be incorporated into such a clinical EEG protocol with minimal disruption, providing patients with a greater chance of demonstrating their awareness when they are incapable of doing so with overt motor behaviors. The preliminary data described here indicate that the current approach is ready for preclinical testing. A large-scale group study must be conducted in order to determine the sensitivity and specificity of this bedside EEG assessment of awareness.

Finally, our single-trial analysis approach also allowed us to correctly identify occasions on which the patient was trying to move his left hand with up to 74% accuracy (unsmoothed, see Methods) from just 1-second of EEG data. These analysis techniques are typical of those used in brain-computer interfaces (BCI) which can provide a form of external control or communication based on mappings of mental states – e.g. trying to move the hand in order to answer ‘Yes’ to a question. The development of techniques for real-time classification of these covert ‘actions’ will not only improve diagnostic accuracy for VS patients, but may also enable some of these patients to communicate, and even gain a level of control over their environment for the first time since their injuries.

## References

[1] Teasdale G, Jennett B (1974) Assessment of coma and impaired consciousness. A practical scale. Lancet 2: 81–84. 4136544 10.1016/s0140-6736(74)91639-0

[2] Giacino JT, Kalmar K, Whyte J (2004) The JFK Coma Recovery Scale-Revised: measurement characteristics and diagnostic utility. Arch Phys Med Rehabil 85: 2020–2029. 15605342 10.1016/j.apmr.2004.02.033

[3] PVS M-STFO (1994) Medical aspects of the persistent vegetative state (1). The Multi-Society Task Force on PVS. N Engl J Med 330: 1499–1508. 7818633 10.1056/NEJM199405263302107

[4] Physicians TRCO (2003) The vegetative state: guidance on diagnosis and management. Clin Med 3: 249–254. 10.7861/clinmedicine.3-3-249 PMC4952451 12848260

[5] Jennett B, Plum F (1972) Persistent vegetative state after brain damage. A syndrome in search of a name. Lancet 1: 734–737. 4111204 10.1016/s0140-6736(72)90242-5

[6] Schnakers C, Vanhaudenhuyse A, Giacino JT, Ventura M, Boly M, et al (2009) Diagnostic accuracy of the vegetative and minimally conscious state: clinical consensus versus standardized neurobehavioral assessment. BMC Neurol 9: 35. 19622138 10.1186/1471-2377-9-35 PMC2718857

[7] Childs NL, Mercer WN, Childs HW (1993) Accuracy of diagnosis of persistent vegetative state. Neurology 43: 1465–1467. 8350997 10.1212/wnl.43.8.1465

[8] Andrews K, Murphy L, Munday R, Littlewood C (1996) Misdiagnosis of the vegetative state: retrospective study in a rehabilitation unit. BMJ (Clinical research ed 313: 13–16. 10.1136/bmj.313.7048.13 PMC2351462 8664760

[9] Cruse D, Chennu S, Chatelle C, Bekinschtein TA, Fernández-Espejo D, et al (2011) Bedside detection of awareness in the vegetative state: a cohort study. Lancet 378: 2088–2094 doi:10.1016/S0140-6736(11)61224-5 22078855 10.1016/S0140-6736(11)61224-5

[10] Cruse D, Chennu S, Chatelle C, Fernández-Espejo D, Bekinschtein TA, et al (2012) Relationship between etiology and covert cognition in the minimally conscious state. Neurology 78: 816–822 doi:10.1212/WNL.0b013e318249f764 22377810 10.1212/WNL.0b013e318249f764 PMC3304945

[11] Owen AM, Coleman MR, Boly M, Davis MH, Laureys S, et al (2006) Detecting awareness in the vegetative state. Science 313: 1402. 16959998 10.1126/science.1130197

[12] Monti MM, Vanhaudenhuyse A, Coleman MR, Boly M, Pickard JD, et al (2010) Willful modulation of brain activity in disorders of consciousness. New England Journal of Medicine 362: 579–589. 20130250 10.1056/NEJMoa0905370

[13] Schnakers C, Perrin F, Schabus M, Majerus S, Ledoux D, et al (2008) Voluntary brain processing in disorders of consciousness. Neurology 71: 1614–1620. 19001251 10.1212/01.wnl.0000334754.15330.69

[14] Pfurtscheller G, Neuper C (1997) Motor imagery activates primary sensorimotor area in humans. Neuroscience Letters 239: 65–68. 9469657 10.1016/s0304-3940(97)00889-6

[15] Pfurtscheller G, Brunner C, Schlogl A, Lopes da Silva FH (2006) Mu rhythm (de)synchronization and EEG single-trial classification of different motor imagery tasks. Neuroimage 31: 153–159 doi:10.1016/j.neuroimage.2005.12.003 16443377 10.1016/j.neuroimage.2005.12.003

[16] Pfurtscheller G, Scherer R, Müller-Putz GR, Lopes da Silva FH (2008) Short-lived brain state after cued motor imagery in naive subjects. European Journal of Neuroscience 28: 1419–1426 doi:10.1111/j.1460-9568.2008.06441.x 18973568 10.1111/j.1460-9568.2008.06441.x

[17] Society ACN (2006) Guideline 1: Minimum Technical Requirements for Performing Clinical Electroencephalography. Journal of Clinical Neurophysiology 23. 10.1097/00004691-200604000-00002 16612222

[18] Bekinschtein TA, Manes FF, Villarreal M, Owen AM, Della-Maggiore V (2011) Functional Imaging Reveals Movement Preparatory Activity in the Vegetative State. Front Hum Neurosci 5. doi:10.3389/fnhum.2011.00005. 10.3389/fnhum.2011.00005 PMC3031991 21441977

[19] Owen AM, Coleman MR, Boly M, Davis MH, Laureys S, et al (2007) Response to Comments on “Detecting Awareness in the Vegetative State.”. Science 315: 1221c–1221c doi:10.1126/science.1135583 10.1126/science.1130197 16959998

[20] Guger C, Edlinger G, Harkam W, Niedermayer I, Pfurtscheller G (2003) How many people are able to operate an EEG-based brain-computer interface (BCI)? Neural Systems and Rehabilitation Engineering, IEEE Transactions on 11: 145–147. 10.1109/TNSRE.2003.814481 12899258

[21] Oostenveld R, Fries P, Maris E, Schoffelen J-M (2011) FieldTrip: Open Source Software for Advanced Analysis of MEG, EEG, and Invasive Electrophysiological Data. Computational Intelligence and Neuroscience 2011: 1–9 doi:10.1155/2011/156869 21253357 10.1155/2011/156869 PMC3021840

[22] Maris E, Oostenveld R (2007) Nonparametric statistical testing of EEG- and MEG-data. J Neurosci Methods 164: 177–190 doi:10.1016/j.jneumeth.2007.03.024 17517438 10.1016/j.jneumeth.2007.03.024

[23] Pfurtscheller G, Lopes da Silva FH (1999) Event-related EEG/MEG synchronization and desynchronization: basic principles. Clinical Neurophysiology 110: 1842–1857. 10576479 10.1016/s1388-2457(99)00141-8

[24] Bishop CM (2006) Pattern recognition and machine learning. New York: Springer. pp.

[25] Maris E (2004) Randomization tests for ERP topographies and whole spatiotemporal data matrices. Psychophysiology 41: 142–151 doi:10.1111/j.1469-8986.2003.00139.x 14693009 10.1111/j.1469-8986.2003.00139.x

[26] Delorme A, Makeig S (2004) EEGLAB: an open source toolbox for analysis of single-trial EEG dynamics including independent component analysis. J Neurosci Methods 134: 9–21. 15102499 10.1016/j.jneumeth.2003.10.009

[27] Pfurtscheller G (1981) Central Beta Rhythm During Sensorimotor Activities in Man. Electroencephalography and Clinical Neurophysiology 51: 253–264. 6163614 10.1016/0013-4694(81)90139-5

[28] Pfurtscheller G, Linortner P, Winkler R, Korisek G, Müller-Putz G (2009) Discrimination of Motor Imagery-Induced EEG Patterns in Patients with Complete Spinal Cord Injury. Computational Intelligence and Neuroscience 2009: 1–6 doi:10.1155/2009/104180 10.1155/2009/104180 PMC2676319 19421415

[29] Pfurtscheller G, Neuper C, Berger J (1994) Source localization using event-related desynchronization (ERD) within the alpha band. Brain Topography 6: 269–275 doi:10.1007/bf01211172 7946926 10.1007/BF01211172

[30] Pfurtscheller G (1992) Event-related synchronization (ERS): an electrophysiological correlate of cortical areas at rest. Electroencephalography and Clinical Neurophysiology 83: 62–69. 1376667 10.1016/0013-4694(92)90133-3

[31] Ritter P, Moosmann M, Villringer A (2009) Rolandic alpha and beta EEG rhythms' strengths are inversely related to fMRI-BOLD signal in primary somatosensory and motor cortex. Hum Brain Mapp 30: 1168–1187 doi:10.1002/hbm.20585 18465747 10.1002/hbm.20585 PMC6870597

[32] Müller-Putz GR, Zimmermann D, Graimann B, Nestinger K, Korisek G, et al (2007) Event-related beta EEG-changes during passive and attempted foot movements in paraplegic patients. Brain Res 1137: 84–91 doi:10.1016/j.brainres.2006.12.052 17229403 10.1016/j.brainres.2006.12.052

[33] Pfurtscheller G, Guger C, Müller G, Krausz G, Neuper C (2000) Brain oscillations control hand orthosis in a tetraplegic. Neuroscience Letters 292: 211–214. 11018314 10.1016/s0304-3940(00)01471-3

[34] Pfurtscheller G, Müller GR, Pfurtscheller J, Gerner HJ, Rupp R (2003) “Thought” – control of functional electrical stimulation to restore hand grasp in a patient with tetraplegia. Neuroscience Letters 351: 33–36 doi:10.1016/S0304-3940(03)00947-9 14550907 10.1016/s0304-3940(03)00947-9

[35] Giacino JT, Ashwal S, Childs NL, Cranford R, Jennett B, et al (2002) The minimally conscious state: definition and diagnostic criteria. Neurology 58: 349–353. 11839831 10.1212/wnl.58.3.349

[36] Pfurtscheller G, Leeb R, Keinrath C, Friedman D (2006) Walking from thought. Brain Res. 10.1016/j.brainres.2005.11.083 16405926

[37] Ball T, Schreiber A, Feige B, Wagner M, Lücking CH, et al (1999) The Role of Higher-Order Motor Areas in Voluntary Movement as Revealed by High-Resolution EEG and fMRI. Neuroimage 10: 682–694. 10600414 10.1006/nimg.1999.0507

[38] Decety J (1996) The neurophysiological basis of motor imagery. Behavioural Brain Research 77: 45–52. 8762158 10.1016/0166-4328(95)00225-1

[39] Leuthold H, Jentzsch I (2001) Neural correlates of advance movement preparation: a dipole source analysis approach. Cognitive Brain Research 12: 207–224. 11587891 10.1016/s0926-6410(01)00052-0

[40] Goldfine AM, Victor JD, Conte MM, Bardin JC, Schiff ND (2011) Determination of awareness in patients with severe brain injury using EEG power spectral analysis. Clinical Neurophysiology 122: 2157–2168. Available:http://www.clinph-journal.com/article/S1388-2457(11)00246-X/abstract. 10.1016/j.clinph.2011.03.022 PMC3162107 21514214

[41] Candelieri A, Cortese MD, Dolce G, Riganello F, Sannita WG (2011) Visual pursuit: within-day variability in the severe disorder of consciousness. J Neurotrauma 28: 2013–2017 doi:10.1089/neu.2011.1885 21770758 10.1089/neu.2011.1885

[42] Bekinschtein TA, Dehaene S, Rohaut B, Tadel F, Cohen L, et al (2009) Neural signature of the conscious processing of auditory regularities. Proc Natl Acad Sci U S A 106: 1672–1677. 19164526 10.1073/pnas.0809667106 PMC2635770

[43] Scott RB, Minati L, Dienes Z, Critchley HD, Seth AK (2011) Detecting conscious awareness from involuntary autonomic responses. Consciousness and Cognition 20: 936–942 doi:10.1016/j.concog.2010.11.009 21130000 10.1016/j.concog.2010.11.009

